# Three-Dimensional Structure of Vertebrate Muscle Z-Band: The Small-Square Lattice Z-Band in Rat Cardiac Muscle

**DOI:** 10.1016/j.jmb.2015.08.018

**Published:** 2015-11-06

**Authors:** Thomas Burgoyne, Edward P. Morris, Pradeep K. Luther

**Affiliations:** 1 University College London, London WC1E 6BT, United Kingdom; 2 Institute of Cancer Research, London SW7 3RP, United Kingdom; 3 Imperial College London, London SW7 2AZ, United Kingdom

**Keywords:** 3D, three-dimensional, 2D, two-dimensional, FT, Fourier transform, electron tomography, electron microscopy, Z-line, Z-disc, α-actinin

## Abstract

The Z-band in vertebrate striated muscle crosslinks actin filaments of opposite polarity from adjoining sarcomeres and transmits tension along myofibrils during muscular contraction. It is also the location of a number of proteins involved in signalling and myofibrillogenesis; mutations in these proteins lead to myopathies. Understanding the high-resolution structure of the Z-band will help us understand its role in muscle contraction and the role of these proteins in the function of muscle. The appearance of the Z-band in transverse-section electron micrographs typically resembles a small-square lattice or a basketweave appearance. In longitudinal sections, the Z-band width varies more with muscle type than species: slow skeletal and cardiac muscles have wider Z-bands than fast skeletal muscles. As the Z-band is periodic, Fourier methods have previously been used for three-dimensional structural analysis. To cope with variations in the periodic structure of the Z-band, we have used subtomogram averaging of tomograms of rat cardiac muscle in which subtomograms are extracted and compared and similar ones are averaged. We show that the Z-band comprises four to six layers of links, presumably α-actinin, linking antiparallel overlapping ends of the actin filaments from the adjoining sarcomeres. The reconstruction shows that the terminal 5–7 nm of the actin filaments within the Z-band is devoid of any α-actinin links and is likely to be the location of capping protein CapZ.

## Highlights

• The vertebrate muscle Z-band connects antiparallel actin filaments from two adjacent sarcomeres.
• Contractile tension is transmitted via α-actinin linking the antiparallel actin filaments.
• We have used electron tomography and subtomogram averaging to investigate the three-dimensional structure of the Z-band in rat cardiac muscle.
• The Z-band is composed of overlapping ends of actin filaments crosslinked by four to six layers of α-actinin.
• The terminal 5–7 nm of the actin filament within the Z-band is devoid of links and may be due to capping protein CapZ.

## Graphical abstract

**Figure:**
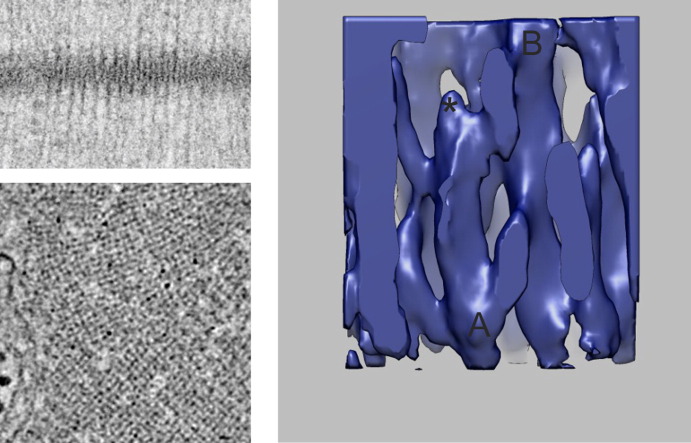

## Introduction

**Fig. 1:**
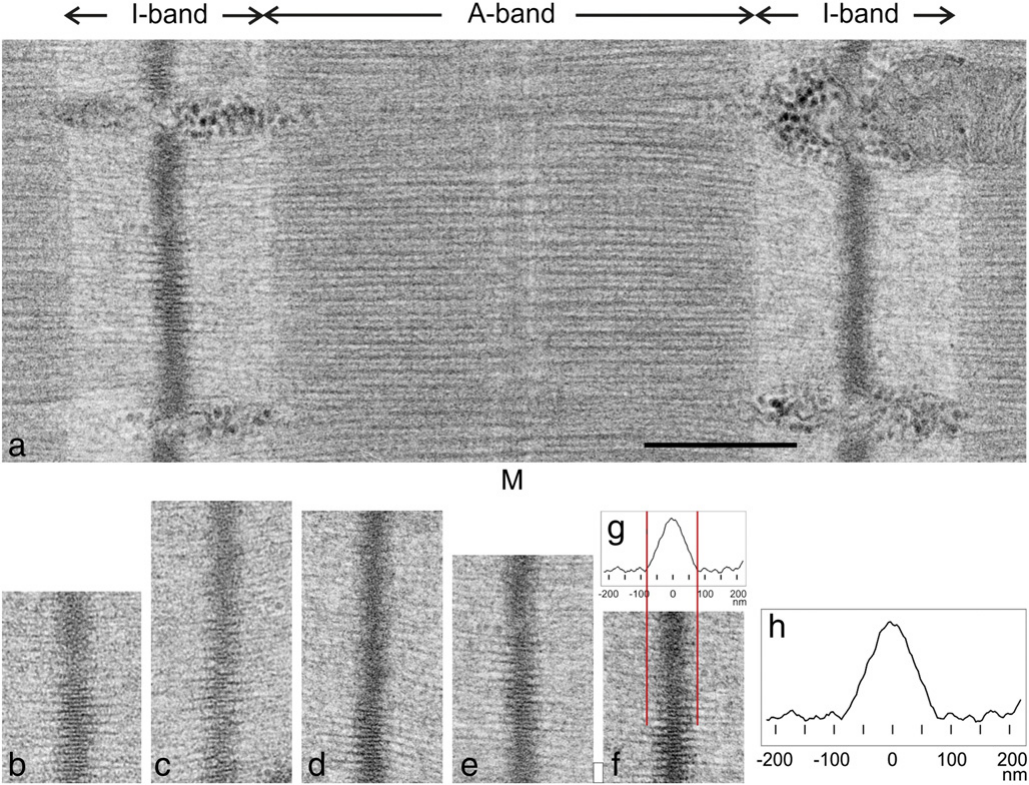
Electron micrographs of the Z-band in longitudinal sections of rat cardiac muscle. (a) A sarcomere showing two Z-bands bisecting the I-bands, A-band and M-band (M). Part of the Z-band on the left shows a clear lattice view of the Z-band tetragonal lattice. The Z-band on the right has a dense fuzzy appearance resulting from a projection off a lattice direction. (b–f) A panel of straight Z-bands showing patches of lattice view. (g and h) Plot profile of the Z-band obtained by averaging the plots of the Z-bands in (b) to (f). The width of the Z-band measured from the base of the plot is about 130 nm. In (g), the plot profile is reduced in size to exactly match the Z-band and I-band in (f), showing how the increase in density in the Z-band matches the projected image. Scale bar in (a) = 0.5 μm (applies to (b) – (f)).

The Z-band (Z-line, Z-disc) defines the boundary of the sarcomere in striated muscle and bisects the I-band of neighbouring sarcomeres (Fig. 1a) [1]. It is one of the two sites crosslinking myofilaments in the sarcomere that serve to maintain interfilament spacing and axial register; the other one is the M-band at the centre of the A-band that crosslinks the myosin filaments. At the Z-band, the barbed ends of opposite polarity actin filaments are crosslinked by α-actinin. Tension generated during muscular contraction is transmitted from sarcomere to sarcomere via the α-actinin links across the Z-band and along the myofibril; hence, the Z-band is directly involved in transmission of tension. In contrast, the bipolar myosin filaments run continuously through the M-band; hence, the M-band is not directly involved in tension transmission.

α-Actinin, the main crosslinking component of the Z-band, is an ~ 35-nm–long, rod-shaped antiparallel homodimer of the spectrin family in which each monomer is composed of an N-terminal actin binding domain, a central rod domain comprising four spectrin repeats and two C-terminal EF-hand domains [2]. The crystal structure of the rod domain [3] and the whole molecule has been solved [4]. It shows that there is an ~ 90° twist between the ends of the rod domain [3], [4]; this is favourable for the assembly of the Z-band. The actin binding domains are quite flexible allowing binding of α-actinin between antiparallel actin filaments (as in the core of the Z-band) and parallel actin filaments [5].

Whereas vertebrate muscle M-bands between fishes and tetrapods (i.e., most land vertebrates) have distinctly different appearances when imaged by electron microscopy [6], [7], Z-bands appear to have fine structure that is less dependent on species but highly dependent on muscle type (skeletal, fast and slow and cardiac). In longitudinal sections, Z-bands have a precisely defined width that varies with muscle type [8], with narrow width (30–50 nm) typical of fast skeletal muscle and with wider width (100–140 nm) typical of slow skeletal and cardiac muscles [9]. The width incorporates a corresponding stack of chevrons or zigzags, with the narrowest forming a single zigzag structure that is found in fish fast myotomal muscle [10], [11] and other fast muscles [mouse musculus vastus lateralis, rabbit psoas, chicken pectoralis (P. Luther, personal observations)]. Previous studies have shown that the ends of the actin filaments overlap in the Z-band and that the amount of overlap is related to the width of the Z-band and the number of layers of α-actinin within the Z-band. The number of layers is 2–4 in fast muscle and 6 in slow muscle [1], [9]. This width precision is disrupted in diseased muscle and with ageing [1].

**Fig. 2:**
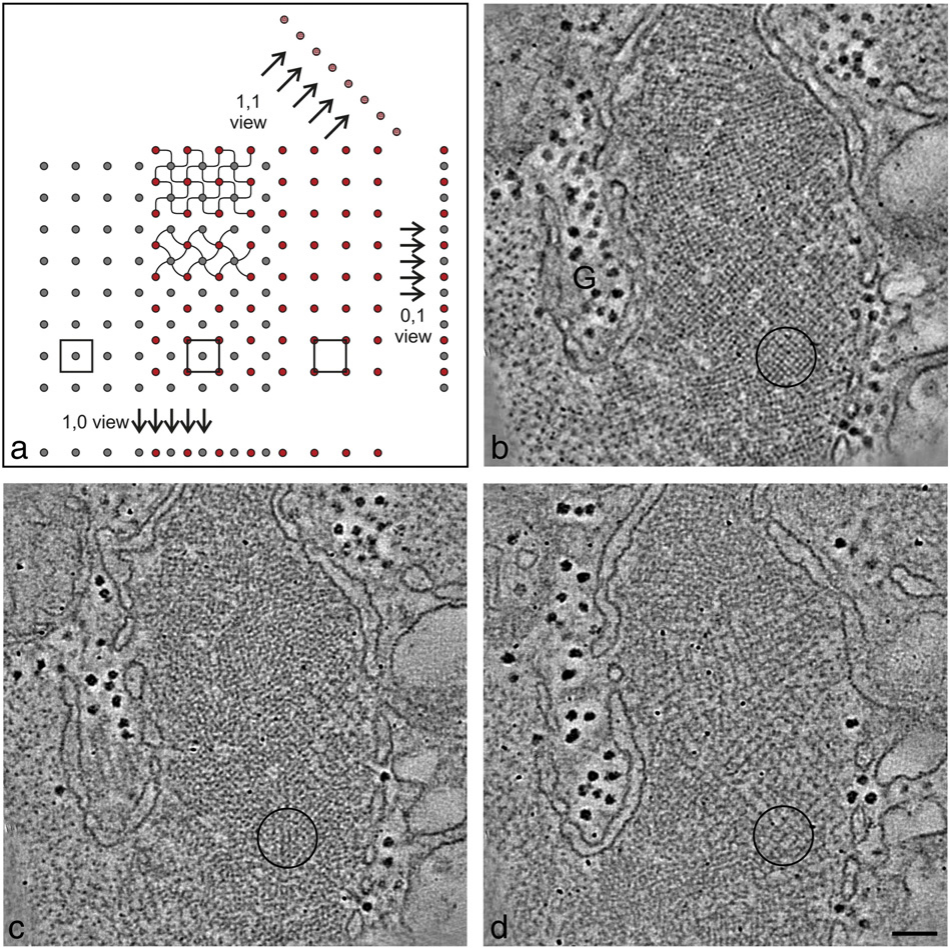
Electron tomography of rat cardiac muscle Z-band. (a) The Z-band tetragonal lattice and nomenclature of lattice views. The main figure illustrates a slightly oblique transverse section with actin filaments (grey) at the left and actin filaments at the right (red) interdigitate in the centre at the Z-band. Small patches of small-square lattice and basketweave Z-bands are shown, the former with sharply bent links and the latter with more gently curved links. Along the bottom, unit cells are outlined at the centre of the Z-band and at either side. Projecting about the major axes gives the lattice views we observe in longitudinal sections, like the 1,0 view and similar orthogonal 0,1 view. Projecting along the diagonal gives the 1,1 view. (b) Electron micrograph of the Z-band region used for the tomography in this study. The Z-band has a clear small-square lattice appearance. Dense glycogen granules (G) are present between the myofibrils. (c) 2D slice of the tomogram near the centre and (d) near one side. As expected from the figure in (a), there is a “small-square” lattice in the central slice (c) due to the overlapping ends of the actin filaments from the adjacent sarcomeres and a large-square lattice at the Z-band periphery (d) due to a single set of filaments from one sarcomere. Corresponding regions showing clear examples of the lattice are encircled in (b)–(d). A movie of the raw tomogram is shown in the supplementary data, Movie S1. Scale bar in (d) = 100 nm (applies to (b) and (c)).

Electron microscopy of transverse sections of vertebrate striated muscle shows that the Z-band has two characteristic appearances; these are described as small-square lattice and basketweave lattice (Fig. 2a) [1], [12], [13], [14], [15]. There is evidence that these appearances are manifestations of the contractile state of the muscle. The former is attributed to relaxed muscle and the latter is attributed to muscle in active contraction [16]. It is believed that the path of α-actinin is altered in the two states and it determines the resulting appearance [14].

An ideal example of the small-square lattice Z-band occurs in the pathological nemaline rods found in patients with the skeletal muscle disease nemaline myopathy. Nemaline rods are ideal for structural analysis of the Z-band as they are composed of extended (up to 1 μm) crystalline Z-bands [17], [18]. In a detailed study of the three-dimensional (3D) structure in nemaline rod Z-bands, Morris *et al*. showed that the path of α-actinin was such that the spectrin rod region runs parallel with the actin filaments and the actin binding domains run perpendicular (Fig. 4a) [19]. This provides a good description of the small-square lattice units that repeat in the direction of the actin filaments, but this does not explain the structure where the actin filaments terminate. Defining the structure of these terminal regions is necessary for a proper understanding of how the Z-band works. This is most appropriately accessed using a Z-band with a small number of repeating units, such as the cardiac Z-band used here.

There have been a small number of studies in the past on the structural determination of the vertebrate muscle Z-band by electron microscopy and 3D reconstruction. In relation to these previous studies, what is special about the present study on the Z-band is as follows. As the Z-band is periodic in nature, Fourier methods are well suited to generate average 3D images of the structure [20], [21]. This was used by Luther [10] to study a narrow Z-band found in fast skeletal muscle showing that it was composed of two layers of α-actinin. Fourier methods were also applied to understand the 3D structure of 3-layer and 6-layer Z-bands [15], [22]. At the other extreme, Morris *et* *al*. reported the structure of the repeating part of the Z-band as found in nemaline rods [19]. A major limitation of the Fourier method as applied in these studies is that averaging is carried out over the whole selected region and this causes smearing in the resulting average since some members (particles, unit cells) can be different.

On the other hand, electron tomography reconstructs the whole selected volume, but as the tomograms are too noisy for direct interpretation of the fine structure, subtomogram averaging is used for extracting particles, correlation matching them by translations and rotations, grading the similarity and then averaging the similar ones [23]. This approach can be applied to objects such as the cardiac Z-band that is made up of repeating 3D motifs containing actin and actin-associated proteins. Here multiple individual 3D motifs are windowed out of the complete tomogram to make up a set of subtomograms, which are then aligned in three dimensions and then averaged to produce subtomogram averages. For Fourier methods, we need a large sample area with excellent order but lattice dislocations in the Z-band mean that ordered patches are small. With subtomogram averaging, we can potentially use all the small patches of partially ordered Z-bands in one or more myofibrils leading to improved signal to noise. In this study, we have used electron tomography and subtomogram averaging for the small-square lattice Z-band in cardiac muscle. We show that it is composed of four to six layers of α-actinin and we show possible location of capping protein CapZ at the ends of the actin filaments within the Z-band.

## Results

### Analysis of the rat cardiac Z-band in longitudinal sections

To understand the 3D structure of the Z-band in a particular muscle, we have found it helpful to study in detail the structure present in both longitudinal and transverse sections and to combine the information gained from both. A typical electron micrograph of a longitudinal section of rat cardiac muscle used for this study is shown in Fig. 1a. The sarcomere has good preservation judged by various criteria: a well-defined A-band with sharp straight edges, a clear straight M-band and well-defined Z-bands. In the Z-band at the left of the image, the middle one-third shows an ordered lattice view resembling a double-sided comb (referred to as the 1,0 view; see Fig. 2a). Here the 3D lattice of the Z-band is viewed along a major axis such that individual layers are directly superimposed in the two-dimensional (2D) projection. The rest of the Z-band and the Z-band on the right have a fuzzy appearance; this is due to the viewing direction of the Z-band lattice no longer lying along a major axis. A gallery of Z-bands from similar longitudinal sections is shown in Fig. 1b–f. These Z-band patches have been selected to include highly straight regions. Each Z-band depicts both 1,0 lattice views and fuzzy views over parts of the full length. Similar to the 1,0 lattice view is the view 90° around the axis, the 0,1 lattice view (see Fig. 2a), and both views are equally likely in a field of sarcomeres. We could not readily distinguish between the 1,0 and 0,1 lattice views in longitudinal sections of this small-square lattice muscle. These views were distinct in our previous studies on basketweave Z-bands [15], [22], possibly because the lattice is slightly larger than small-square lattice Z-bands [12].

To measure the lattice size of the Z-band, we first examined the Fourier transforms (FTs) of half A-bands in longitudinal sections in which the 43-nm crossbridge reflection was clearly present and we used this to provide an internal calibration. (References to lattice in this study are only for the lateral arrangement of the actin filaments within the Z-band.) The lattice size was measured as 29 ± 2 nm. This is larger than the value of 24 nm of the reflection that is ascribed to the small-square lattice Z-band in X-ray patterns of relaxed muscle [12], [24]. This discrepancy is puzzling. Dimensional changes especially shrinkage occur during the processing for electron microscopy and during electron microscopy viewing [25], [26]. We would expect similar shrinkage effects in the electron microscope images of the A-band crossbridge region and the Z-band. The crossbridge repeat is known from prominent spots in the X-ray diffraction of live muscle [27].

The occurrence of ordered lattice views over only limited regions of individual Z-bands in the longitudinal sections can be explained by distortions or dislocations in the lattice (discussed below). Projection of the 2D density in the images perpendicular to the sarcomere axis allows the generation of a density profile (Fig. 1g and h). From the thickness of the longitudinal section used, ~ 100 nm, there are about 3 unit cells of the 29-nm lattice spacing in the depth of the section; hence, the density profile is substantially independent of the axial orientation of the sarcomere, whether the Z-band presents a lattice view or a fuzzy view. The plot profiles of the regions shown in Fig. 1b–f were aligned by cross-correlation and their averaged density profile is shown in Fig. 1g and h. This shows that the total width of the Z-band is about 130 nm measured from near the base of the graph. In our previous studies relating Z-band width with the number of α-actinin layers contributing to it [9], we found that six α-actinin layers can occur in ~ 130-nm-wide Z-bands. Hence, six α-actinin layers are a good estimate for the composition of rat cardiac Z-band.

### Electron tomography of the Z-band in rat cardiac muscle

The basis for the 3D structure of the vertebrate muscle Z-band is illustrated in Fig. 2a. The figure shows a schematic cross-section of a slightly oblique Z-band and I-band, with actin filaments of one orientation shown in grey-filled circles and those with the opposite orientation shown in red. Unit cells in the centre of the Z-band and at either side are outlined. The diagram illustrates that the lattices of the actins from the adjoining sarcomeres have a half unit-cell offset between them. The main lattice views seen in longitudinal sections are the 1,0 and 0,1 views or lattice projections. Projecting about the diagonal of the lattice gives the 1,1 view.

Tomograms of rat cardiac muscle were generated by electron microscopy of transverse sections of ~ 100 nm thickness. This thickness includes about 2.6 actin half repeats of 76 nm (38 nm). One tomogram is illustrated in Fig. 2b–d. The area selected for the tomography (Fig. 2b) comprises Z-band regions with well-defined small-square lattices; a clear example is encircled. In common with Z-bands in this muscle, this Z-band region does not have a continuous lattice across the myofibril; it is characterised by lattice dislocations across the face of the myofibril. As mentioned earlier, in longitudinal sections, this can result in ordered comb-like lattice views adjacent to fuzzy Z-bands. Two tilt series perpendicular to each other were recorded between the range + 60° and − 60° in steps of 2°. The back projections and combination of the dual-axis tilt series were computed using IMOD software [28]. Gold particles of 10 nm diameter were used for alignment of the tilt series. Two slices of the tomogram are shown: one near the centre of the tomogram (Fig. 2c) and one near the edge (Fig. 2d). Prominent glycogen granules (G) outside the myofibrils are seen (Fig. 2b–d). In the slice near the centre of the Z-band (Fig. 2c), the region associated with well-preserved small-square lattice in the untilted image (Fig. 2b, circled) is characterised by closely spaced actin filament cross-sections. At the edge of the tomogram, the equivalent region (Fig. 2d, circled) shows a large-square lattice indicating that the region is located at the periphery of the Z-band where there are actin filaments from one side of the Z-band only and there is no overlap of actin filaments from the adjacent sarcomere. A movie of the tomogram traversing from one edge of the tomogram to the other is shown in Supplementary Movie 1. In Fig. 2c and the tomogram movie, some connections can be seen between the actin filaments but they are irregular and noisy, typical of biological structure tomograms. In order to characterise such connections, it is necessary to average equivalent motifs so as to improve the signal-to-noise ratio. We estimate that the resolution in the tomogram is about 8 nm measured from the spots in the FTs of the electron micrographs.

### Subtomogram averaging of the tomograms

**Fig. 3:**
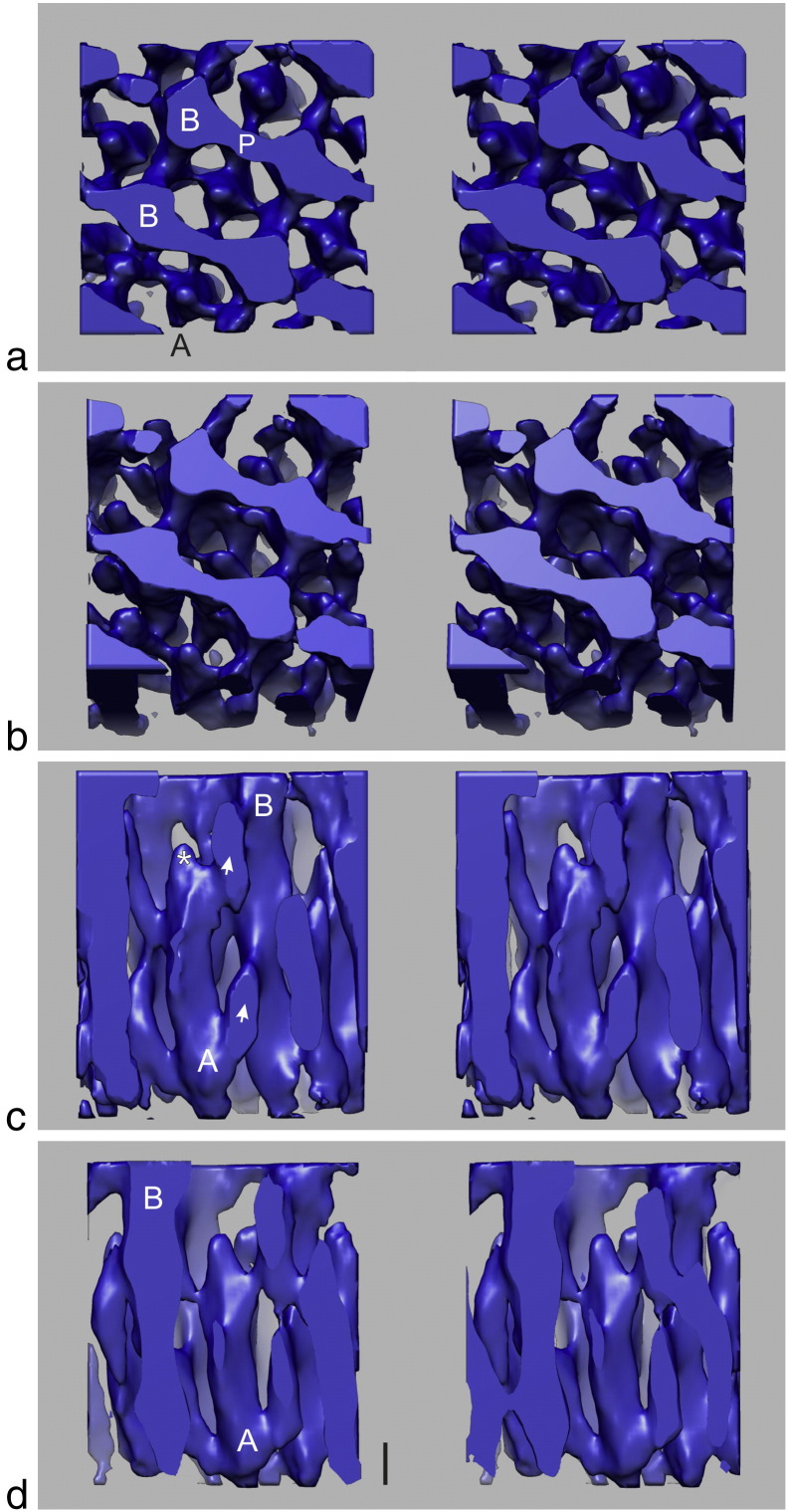
Surface-rendered stereo images of the small-square lattice Z-band in rat cardiac muscle following subtomogram averaging. Examples of the two sets of actin filaments of opposite polarity from sarcomeres below and above are labelled A and B, respectively. (a) Plan view. Links between the filaments are seen that run parallel with the lattice as found in small-square lattice Z-bands. (b) Oblique view showing more detail of the filaments and links. (c) Side view showing A and B polarity actin filaments with clear links (arrowheads) between them at a nominal periodicity of 38 nm. The centre of the Z-band is not at the centre of the tomogram; hence, we see the end of the A-filaments as they terminate inside the tomogram but the B-filaments are clipped at the bottom face of the tomogram. The terminal region of the A-filament marked with an asterisk (*) is devoid of links. We believe that this region may represent capping protein CapZ. (d) As (c) but 1 unit cell deeper into the average tomogram showing another clear example of an A-filament with a bare terminus region. In (a) and (b), prominent links (labelled P) occur between the same polarity B-filaments; these were called polar links in our previous study [10]. Scale bar in (c) and (d) represents 10 nm in vertical direction.

Subtomogram averaging of the tomograms was performed using PEET (*p*article *e*stimation for *e*lectron *t*omography) software running through ETOMO package [29]. Unit cells corresponding to the small-square lattice (Fig. 2a) were marked using IMOD and typically about 90 regions centred on individual unit cells were extracted for alignment and averaging. The average for one of the tomograms is shown in Fig. 3 and in Supplementary Movie 2. It is cuboid in shape and encloses about 2 unit cells of the Z-band lattice. To aid in describing the tomograms, we will refer to actin filaments originating from one side of the Z-band as A-actin filaments and those from the opposite side as B-actin filaments. As shown in Fig. 3c, the A-filaments originate from the lower sarcomere and terminate inside the 3D volume near the opposite face. The B-filaments originate from the upper sarcomere and these filaments do not terminate inside the 3D volume; they continue to the lower face where they are clipped. Thus, the centre of the Z-band is offset relative to the centre of the section. Figure 3c reveals clear connecting density from the A-filaments to the neighbouring antiparallel B-filaments (marked with arrowheads); this density can be ascribed to α-actinin. The origins of the linking density on either side of the A-filament are not at the same axial level but are offset by about 2–5 nm. This is consistent with the symmetry of actin filament, as discussed in more detail later. For the total number of links emanating from a single filament and connecting to the opposite polarity filaments, we can clearly count three links. However the tomogram volume does not include the whole Z-band. The end part of filament A marked with an asterisk (*) in Fig. 3c is about 5–7 nm long and it is devoid of any connecting density. This region is likely to contain the actin capping protein CapZ [30], [31]. Another clear feature shown in Fig. 3a and b is the linking density (P) between the same-polarity B-filaments at the level of the Z-band periphery. This density is perpendicular to the filaments and runs in one direction only; similar links were observed by Luther [10] who referred to them as polar links.

### Modelling the rat cardiac Z-band

**Fig. 4:**
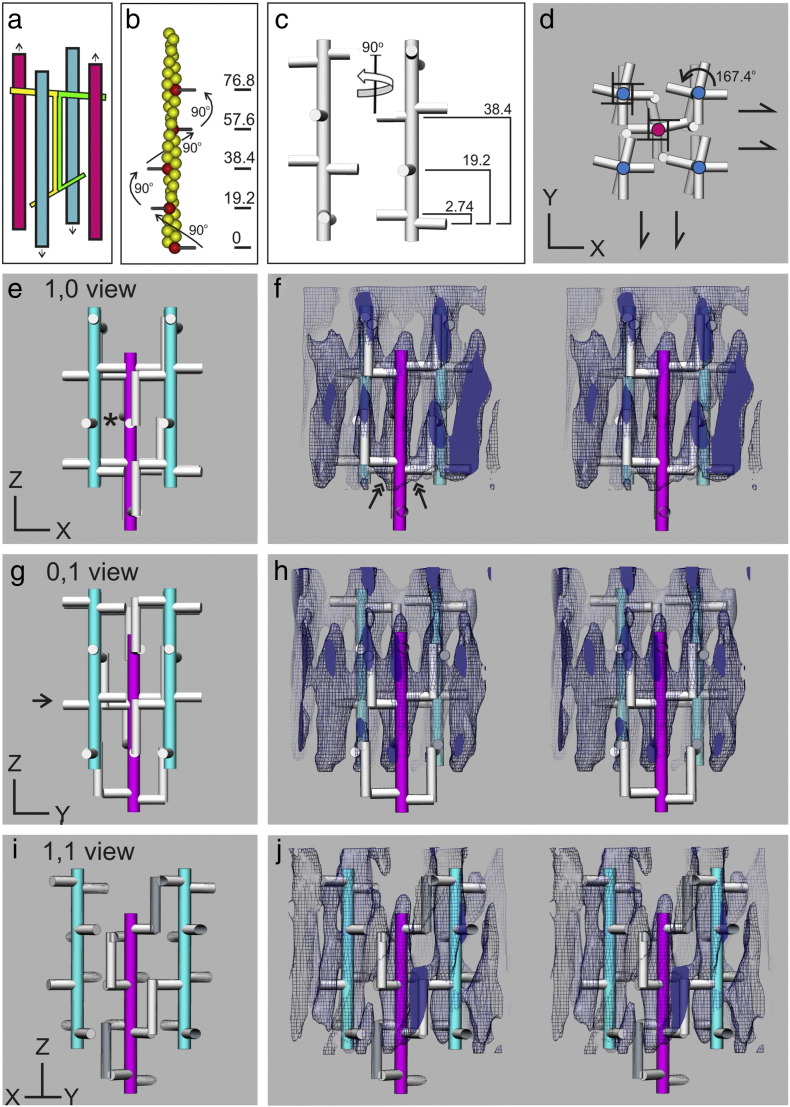
Interpretation of the rat cardiac Z-band tomogram by modelling. (a) Schematic model of the Z-band motif for small-square lattice Z-bands proposed by Morris *etal*. (1990) composed of two pairs of antiparallel actin filaments (magenta and cyan) linked by two α-actinin dimers shown in yellow and green. α-Actinin is a homodimer comprising a central axial rod domain (parallel with actin) and transverse actin binding domains (“struts”) with a relative twist of 90°. (b) Helical actin filament in which the monomers are related by an axial rise of 2.74 nm and a rotation of 167.1° so that every seventh actin subunit (red) is spaced 19.2 nm apart and rotated by 90° forming 4_3_ screw symmetry-related binding sites for α-actinin. (c) Two sets of such α-actinin binding sites related to each other by 2.74 nm axial translation and 167.1° rotation about the filament axis form the basis of the repeating motif of the small-square Z-band lattice. (d) Overall symmetry of the repeating motif of the small-square Z-band illustrated with an end view of a schematic model comprising one up filament (magenta) and four down filaments (cyan) and associated α-actinin links (white). The 4_3_ screw symmetry axes of the actin filaments combine with 2_1_ screw symmetry axes midway between the two sets of filaments resulting an overall *P*4_3_2_1_2 symmetry. (e–j) Comparison of schematic model with the protein density of the subtomogram average in 1,0 (e and f), 0,1 (g and h) and 1,1 (i and j) views. Schematic models are shown separately (e, g and i) and in stereo view within the protein density (f, h and j). In (f), clear links are seen between the antiparallel filaments (double-headed arrows). In the 1,1 view (i and j), α-actinins show the 90° relative twist of the struts at each end of the rod. (f, h and j). This figure is shown as Movie S4 in the supplementary data.

To understand the averaged tomograms further, we constructed a simple model based on the Morris scheme [19] for two pairs of oppositely oriented filaments and their links (Fig. 4a). Each α-actinin molecule is simplified into an axial rod that bends at right angles at each end to give actin binding struts (AB). There is a twist of 90° between the two AB struts as found in the crystal structures of the rod [3], [32]. We have constructed the model with four pairs (layers) of α-actinin molecules emanating from each actin filament end at the Z-band (Fig. 4c) although there may be six layers in the rat cardiac Z-band. Within the Z-band, actin filaments have helical symmetry 28/13 [19], [33] (i.e., 28 subunits in 13 turns of helix) and each pair of nearly diametrically opposed α-actinin molecules has a relative angular shift of ~ 167° and an axial shift of 2.76 nm (Fig. 4c). We have used this filament to construct a unit cell of the Z-band comprising one A-filament and four B-filaments (Fig. 4d). We joined the AB struts with axial rods as in the Morris scheme (Fig. 4e, g and i). To clarify the model, we have coloured in the actin filaments originating from each side of the Z-band, magenta for the bottom A-filaments and cyan for the upper B-filaments. Different views of the model along with the axes are shown in Fig. 4d, e, g and i and in Supplementary Movie 3; (d) shows the transverse view, (e) shows the 1,0 view, (g) shows the 0,1 view and (i) shows the 1,1 view. An important distinction between the 1,0 and 0,1 view is that the former has central hollows [labelled with an asterisk (*) in (e)] whereas the latter 0,1 view has the AB struts forming a transverse density [arrow in (g)]. In Fig. 4f, h and j and Supplementary Movie 4, we attempt to fit the 3D reconstruction shown in Fig. 3 with the model. The 1,0 and 0,1 views in Fig. 4f and h match the central hollow region in the former and the central transverse density in the latter. Careful examination of the 1,1 view (j) shows some well-matched features of the reconstruction with the model. Note especially the links between the A-filament and the B-filament in (f) marked with double-headed arrows. We note that the links between antiparallel actins are inclined at acute angles whereas the struts that we have modelled are perpendicular; the acute angle is probably due to the low resolution of the reconstruction.

## Discussion

The primary function of the Z-band is the transmission of tension from one sarcomere to the next. As actin filaments are not continuous through the Z-band, the tension has to be transmitted through the Z-band assembly; hence, understanding the 3D structure is important. Here we have carried out electron tomography of the Z-band in rat cardiac muscle using ~ 100-nm transverse sections of the Z-band. As pointed out in the introduction section, the structure of vertebrate muscle Z-bands varies more with muscle type than species. The rat cardiac Z-band studied here is wide, typical of mammalian cardiac and slow skeletal muscles, and in transverse sections, it has a small-square lattice appearance. Typical of biological samples, the raw tomograms were very noisy. As the Z-band is periodic, there are many copies of the unit cells; hence, we can apply subtomogram averaging using suitable software, for example, PEET. The averaged tomogram (Fig. 3) shows clear actin filaments and connecting links that we ascribe to α-actinin. The origins of the links on one actin filament appear to follow 4-fold screw symmetry; that is, symmetry-related positions occur in one-fourth repeat along the filament (19.2 nm) and are rotated by 90° (Fig. 4b). At the 8-nm resolution of the reconstruction, we do not see subunits of actin or further details of α-actinin. However, we are pleased that the tomogram reveals the terminal 5–7 nm of the actin filament as bare and that the first α-actinin linking density starts after this region. We believe that this terminal 5–7 nm is the location of CapZ. Improvement in resolution in future studies will allow the fitting of crystal structures of actin [34], CapZ [30] and the recently solved α-actinin [4].

### Width of the Z-band in longitudinal sections of rat cardiac muscle

In our previous studies [9], [22], we showed that we can make a good estimate of the number of layers of α-actinin comprising the Z-band in that muscle by examining the Z-band of a muscle in longitudinal sections. We showed the appearance of the Z-band in 2-layer, 3-layer, 4-layer and 6-layer Z-bands and we proposed how we can compare the images of these Z-bands with those of a new sample in order to determine the composition of the new sample. Firstly, in a Z-band with an even number of layers of α-actinin, the image of the Z-band in a longitudinal 1,0 view has a comb-like appearance on both sides of the Z-band, whereas for Z-bands with an odd number of layers, the comb-like appearance is only on one side as shown for a 3-layer Z-band in fish fin muscle [9], [22]. Here in rat cardiac muscle Z-band, the comb-like appearance is on both sides (Fig. 1); hence, the number of layers must be even. In our previous studies, we showed that a Z-band of width ~ 130 nm comprises six layers of α-actinin [15]. As we have measured the same width in this study, we infer that the number of layers of α-actinin in rat cardiac muscle must be 6. In the present study, the section that we have used for the tomography included only part of the Z-band in its width and we have identified only three layers of α-actinin. From the even number due to the comb-like appearance, the number of layers is therefore likely to be 4 or 6. Tomography will have to be carried out for a longitudinal section or a sufficiently thick transverse section in order to confirm the number of layers of α-actinin links within the rat cardiac Z-band.

It is worth speculating on reasons why the Z-band in fast-twitch muscles is narrow with few layers of α-actinin and that in slow-twitch and cardiac muscles is wide with higher number of layers of α-actinin. The forces generated during contraction of fast-twitch muscle are higher and faster than the latter; thus, we would expect the narrow Z-bands in these muscles to be more prone to distortions and axial shifts of the filaments. With more layers of α-actinin and slower contraction in slow and cardiac muscles, we would expect the Z-band to be more rigid and better able to maintain its structure than fast-twitch muscle. It is not clear why we need more rigid and stable Z-bands in slow-twitch and cardiac muscles than in fast-twitch muscles.

It is important to note that the widths of the Z-bands in the longitudinal sections of this muscle were quite constant (Fig. 1); we especially note that there were not any large deviations resulting in abnormally wide or irregular Z-bands that follow insult: a hallmark of healthy muscle is the presence of Z-bands with constant width [1].

### Comment on a previous 3D tomographic reconstruction of vertebrate muscle Z-band

A previous tomographic reconstruction (i.e., whole volume reconstruction) of vertebrate muscle Z-band by Schroeter *et* *al*. [35] examined the Z-band in slow skeletal soleus muscle of rat. They analysed the 3D structure of the Z-band in the tomogram directly by examining prominent features such as actin filaments and other filamentous objects. This approach is the only method of structural analysis for large heterogeneous samples (e.g., mitochondria) in which every member is different. Such objects cannot be directly averaged and we have to make judgement about the overall structure by visually comparing common features of several different tomograms. Schroeter *et* *al*. identified Z-band components such as actin filaments and connecting struts in the tomogram directly [35]. However, electron tomograms of biological samples are typically noisy and averaging greatly helps to improve visibility. Since the Schroeter study lacks averaging, it is hard to evaluate the mean structure underlying the particular Z-band. In the current study, we have used tomography followed by subtomogram averaging of the unit cells in the Z-band to improve the visibility of the fine 3D structure of the Z-band.

### Previous 3D reconstructions of the Z-band based on Fourier averaging methods

To understand the structure of macromolecular samples organised in arrays, Fourier methods are ideal as they provide information about the average structure. A prime example is crystallographic analysis of macromolecules using X-ray diffraction of crystals that are composed of 3D arrays of macromolecules. For electron microscopy, the pioneering work of Henderson and Unwin laid the foundation of methods to use Fourier analysis in order to unravel the 3D structure of proteins organised in 2D protein arrays [36]. Luther applied these tilt series methods to generate the 3D reconstruction of the Z-band in fish body white muscle [10]. This fast skeletal muscle has a narrow Z-band that appears in longitudinal sections as a single zigzag connecting the ends of the antiparallel actin filaments. The 3D reconstruction showed that there were two layers of α-actinin separated by ~ 19 nm with one layer orthogonal to the other. The reconstruction also showed that, at the periphery of the Z-disc, there were perpendicular links (“polar links”) between actin filaments of the same polarity. The linking of parallel actin filaments by α-actinin has been demonstrated in isolated filaments [5]. In this study, we have observed similar density in rat cardiac muscle (Fig. 3a and b). Whether this linking density is α-actinin or one of the several proteins found in the Z-band will require immunolabelling analysis.

For Fourier averaging methods, the larger the ordered array is, the better the quality of the final average is. For Z-bands, large ordered 2D arrays occur in pathological muscle affected with nemaline myopathy. The 3D structure of the Z-band in nemaline rods was studied by Morris *et* *al*. [19]. Their Fourier-based methods involved recording particular lattice views (1,0, 1,1 and 2,1) in thin longitudinal resin sections. The central slice theorem of Fourier analysis states that the FT of a 2D projection of a section of a 3D sample is a central slice of the 3D FT. Morris *et* *al*. used the FTs of the three views and the symmetry of the Z-band to generate the 3D FT and hence the 3D map of the nemaline Z-band structure.

The Morris *et* *al*. study showed the structure of the repetitive part of the Z-band present in nemaline rods. Normal Z-bands are much narrower and the structure of the nonrepeating parts similar to the periphery is missing from the Morris study. Luther *et* *al*. used the Morris *et* *al*. method to generate the 3D structure of the Z-band in two different muscles that were found to comprise three and six layers of α-actinin [15], [22]. In contrast to the Fourier methods, the tomography and subtomogram averaging used in the present study allows for variations in the fine structure between different unit cells and only matching unit cells are averaged. Nevertheless, valuable insight in the 3D structure of Z-bands was obtained from the Morris and Luther studies employing Fourier methods.

### Small-square and basketweave Z-bands in striated muscle

In transverse sections, the vertebrate muscle Z-band has two characteristic appearances described as basketweave and small-square lattice. Here the structure is clearly that of the small-square lattice type (Fig. 2a). This justifies the use of the Morris scheme [19] for the path of α-actinin in a small-square lattice Z-band in modelling the rat Z-band tomogram. The other view is basketweave that has been studied by Luther *et* *al*. [15], [22]. The modelling by Luther *et* *al*. accounts well for the features in the basketweave Z-bands of these muscles [15], [22]. There is considerable evidence that that two forms of lattice can be generated by the state of the muscle, the small-square form occurring in relaxed muscle and transforming to the basketweave form in actively contracting muscle [13], [16]. The mechanism of the transition from small square to basketweave is thought to involve a change from a nearly right angle bend to a gentler curve of the rod and actin binding domain of α-actinin (Fig. 2a) accompanied by an expansion of the lattice by about 10% [12].

### The Z-band as a multiprotein hub

There is great interest in the Z-band currently as a plethora of proteins have been found here in the last two decades. These proteins are probably not part of the contractile chain but have other roles especially in signalling, mechanosensation and mechanotransduction. Excellent reviews have been written on our current knowledge of these proteins and their possible roles [37], [38], [39], [40].

Our identification of α-actinin and CapZ has important medical relevance. Recent work [41] has shown that mutations in α-actinin can lead to cardiac disease. They found that mutations in the actin binding domain in α-actinin can lead to hypertrophic cardiomyopathy and that mutations in the spectrin domain can lead to dilated cardiomyopathy and arrhythmias. They suggested that, in the former case, the binding to actin may be affected leading to altered contractile properties. In the latter case, the binding of the noncontractile proteins may be affected. Secondly, Yang and Pyle found that reduction in the amount of CapZ is cardioprotective against ischemia/reperfusion injury [42]. Improvement in the resolution of Z-band tomography would enable the study of the effects of mutations in these proteins.

## Materials and Methods

### Sample preparation

Rat papillary muscle was dissected under oxygenated Krebs solution with 30 mM 2,3-butanedione monoxime (to ensure relaxed state of the muscle), pinned to Sylgard gel in a Petri dish and fixed in 3% glutaraldehyde in the Krebs buffer for 3 h. After rinsing, the muscle was fixed in 1% aqueous osmium tetroxide, dehydrated in acetone series and embedded in Araldite epoxy resin. Thin ~ 100-nm sections were cut with a Reichert Ultracut-E ultramicrotome, picked on thin bar hexagonal grids†, labelled with 10-nm gold particles and stained with 2% uranyl acetate followed by Reynold's lead citrate. The gold particles were prepared by the method of Slot and Geuze [43].

### Electron microscopy and tomography

The sections were examined in a JEOL 1200 EX electron microscope and photographed using a Tietz Fastscan-114 1024 × 1024 CCD camera operated through EM Menu4 software‡. For the tomography, JEOL 1200 and an FEI CM200 electron microscopes were used. Suitable selected regions were photographed at a magnification of 15,000 × (JEOL) or of 27,500 × (CM200). Tilt series about two orthogonal axes were recorded from − 60° to + 60° in steps of 2°. The tilt series images were aligned and back-projected using IMOD software [28]. To obtain mean 3D images, we carried subtomogram averaging out using PEET software running under the IMOD environment [29].

### Modelling of Z-band

3D modelling of the Z-band was performed using SketchUp§. Rods (5 nm diameter) representing actin filaments were placed on a square lattice of size 30 nm (Fig. 4c and d). The origins of α-actinin on the actin filaments were calculated for 28/13 symmetry of actin (28 subunits in 13 turns of the helix; rotation of 167.1° and axial rise of 2.74 nm per monomer). This means that every seventh actin monomer has a rotation of 90° and axial rise of 19.2 nm (shown in red in Fig. 4b).

The following are the supplementary material to this article.

Supplementary Movie 1 Tomogram of Z-band of rat cardiac muscle.
Movie traversing 2D slices through the depth of one of the dual-axis tomograms of the Z-band in rat cardiac muscle. Glycogen granules and the membrane boundaries are prominent outside the myofibrils. Arrays of thin filament cross-sections arranged in small-square lattices occupy the central myofibril over most of the tomogram. At the outer edges of the tomogram (i.e., near the start and end of the movie), only the filaments from one sarcomere are present and here they have larger square lattice. There is ample linking density between the actin filaments but this is highly variable; hence, averaging is essential. We suggest that the movies in this study be viewed with a player such as Apple QuickTime that allows frame-by-frame advancement that can be achieved by pressing either the left or right arrows on the keyboard.

Supplementary Movie 2 Movie showing the details of the rendered subvolume of the tomogram following subtomogram averaging. The movie starts in plan view and then shows the front view in two parts: the first part displays the whole subvolume and the second part has a narrow depth and depth-shading to enhance the visualisation of the actin filaments and links. Two sets of filaments are present entering the Z-band from the lower and upper sarcomeres. Between the two sets of filaments are prominent links that we ascribe to α-actinin.

Supplementary Movie 3 Movie illustrating the Z-band model described in Fig. 4. A magenta actin filament from the lower sarcomere is surrounded by four cyan actin filaments from the upper sarcomere. The two sets of actin filaments are connected by schematic α-actinin molecules comprising an axial rod with transverse struts at each end with a 90° relative twist. Only four sets of links are shown although there may be six in this Z-band.

Supplementary Movie 4 Movie showing a thin slab of the model superimposed on a semitransparent thin slab of the averaged tomogram. By stepping through the movie frame by frame (e.g., using QuickTime player), there is excellent match at some timepoints between the model and the tomogram.

## Footnotes

† www.agarscientific.com.

‡ www.tvips.com.

§ www.sketchup.com.
