# Supplementary figures and images for: Three-Dimensional Structure of Vertebrate Muscle Z-Band: The Small-Square Lattice Z-Band in Rat Cardiac Muscle
Source: J Mol Biol. 2015 Nov 6;427(22):3527–37. doi: 10.1016/j.jmb.2015.08.018 (PMC4641244; doi:10.1016/j.jmb.2015.08.018)

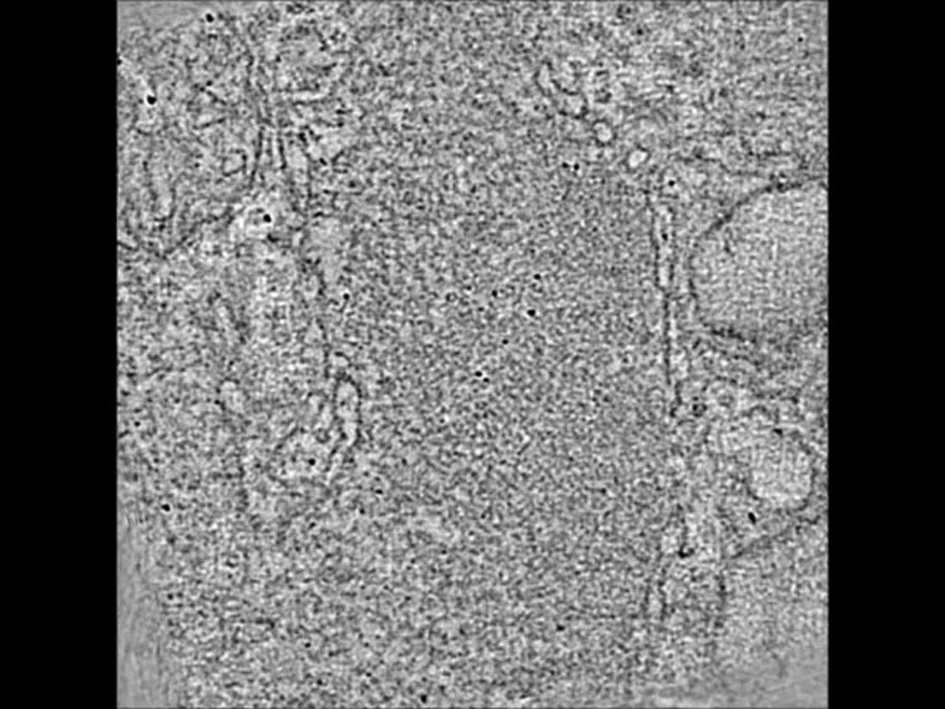

Supplement: Supplementary Movie 1 — Tomogram of Z-band of rat cardiac muscle. Movie traversing 2D slices through the depth of one of the dual-axis tomograms of the Z-band in rat cardiac muscle. Glycogen granules and the membrane boundaries are prominent outside the myofibrils. Arrays of thin filament cross-sections arranged in small-square lattices occupy the central myofibril over most of the tomogram. At the outer edges of the tomogram (i.e., near the start and end of the movie), only the filaments from one sarcomere are present and here they have larger square lattice. There is ample linking density between the actin filaments but this is highly variable; hence, averaging is essential. We suggest that the movies in this study be viewed with a player such as Apple QuickTime that allows frame-by-frame advancement that can be achieved by pressing either the left or right arrows on the keyboard. [file mmc1.jpg]

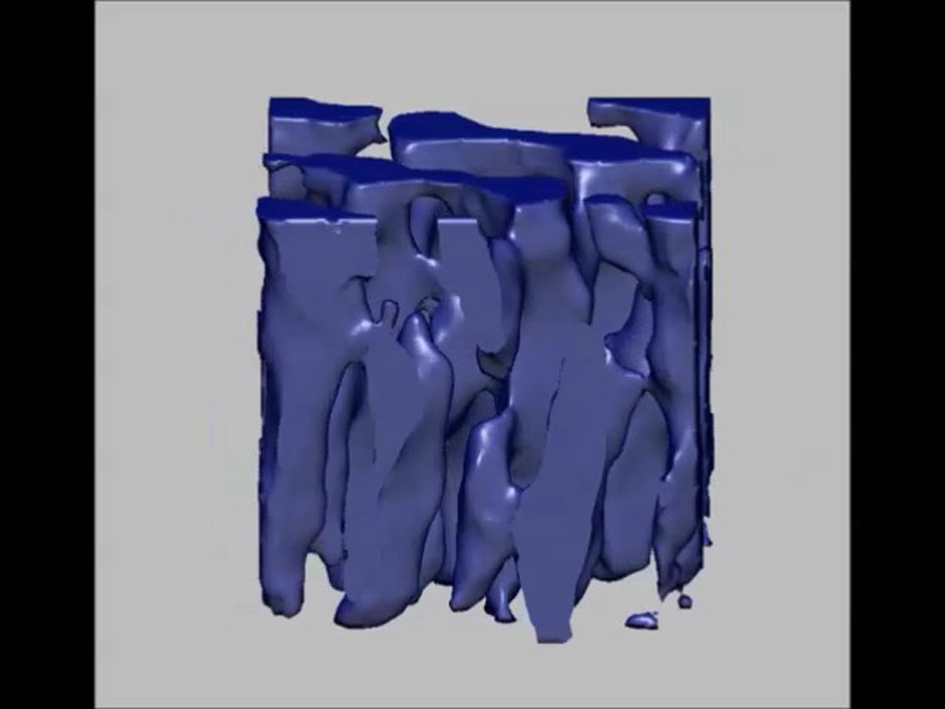

Supplement: Supplementary Movie 2 — Movie showing the details of the rendered subvolume of the tomogram following subtomogram averaging. The movie starts in plan view and then shows the front view in two parts: the first part displays the whole subvolume and the second part has a narrow depth and depth-shading to enhance the visualisation of the actin filaments and links. Two sets of filaments are present entering the Z-band from the lower and upper sarcomeres. Between the two sets of filaments are prominent links that we ascribe to α-actinin. [file mmc2.jpg]

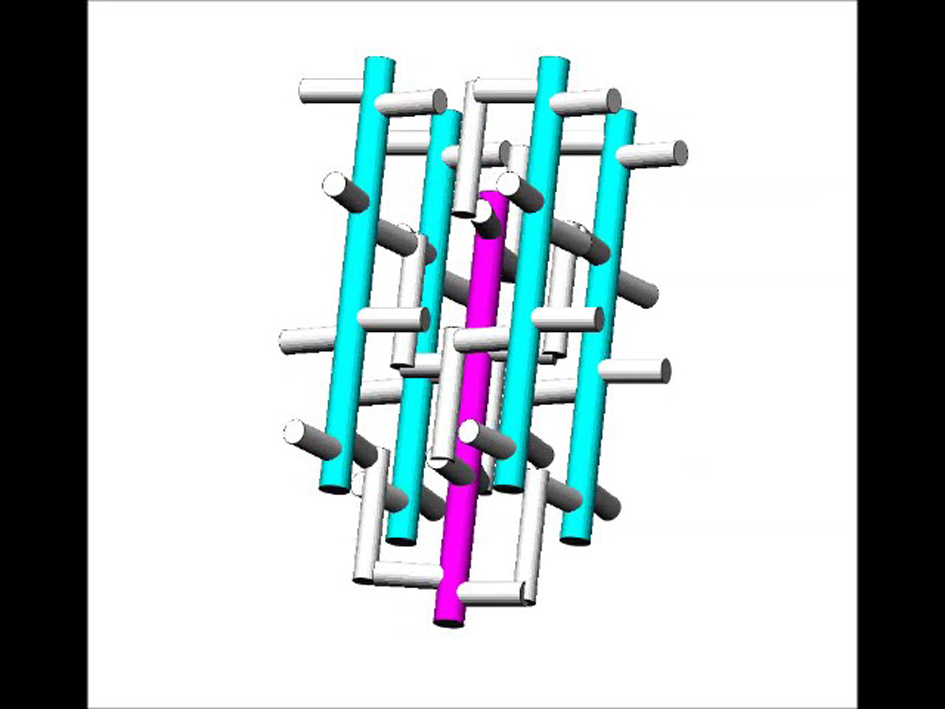

Supplement: Supplementary Movie 3 — Movie illustrating the Z-band model described in Fig. 4. A magenta actin filament from the lower sarcomere is surrounded by four cyan actin filaments from the upper sarcomere. The two sets of actin filaments are connected by schematic α-actinin molecules comprising an axial rod with transverse struts at each end with a 90° relative twist. Only four sets of links are shown although there may be six in this Z-band. [file mmc3.jpg]

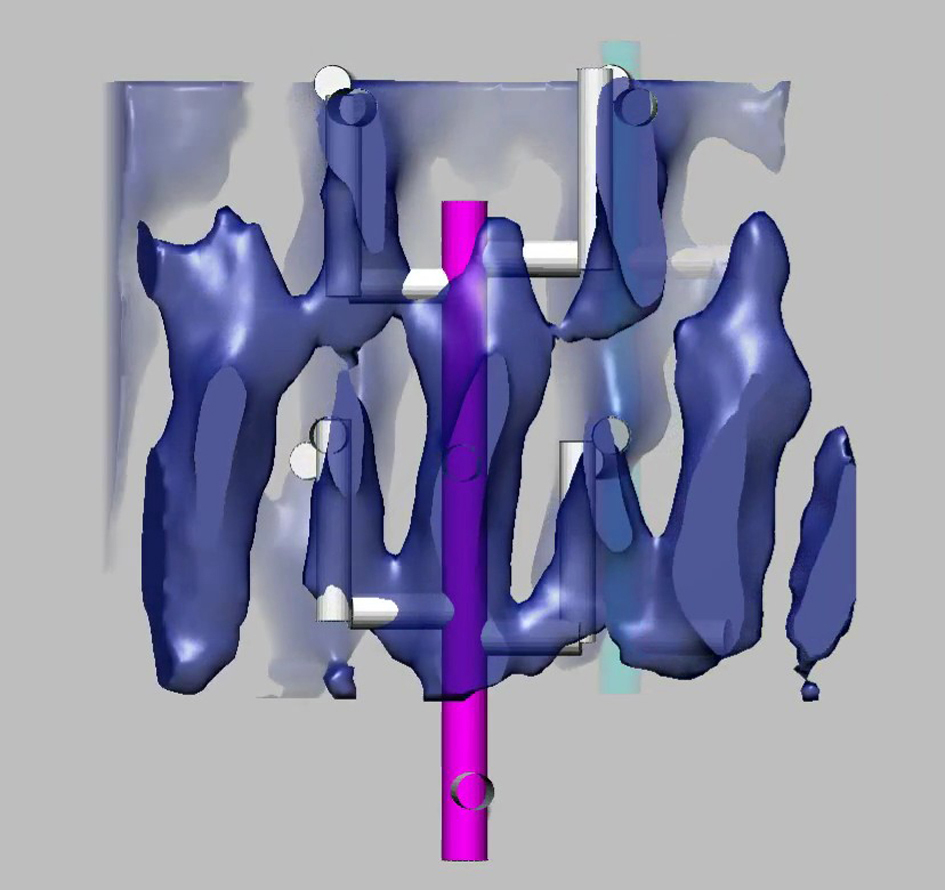

Supplement: Supplementary Movie 4 — Movie showing a thin slab of the model superimposed on a semitransparent thin slab of the averaged tomogram. By stepping through the movie frame by frame (e.g., using QuickTime player), there is excellent match at some timepoints between the model and the tomogram. [file mmc4.jpg]
